# Small Extracellular Vesicles Derived from Cord Blood Plasma and Placental Mesenchymal Stem Cells Attenuate Acute Lung Injury Induced by Lipopolysaccharide (LPS)

**DOI:** 10.3390/ijms26010075

**Published:** 2024-12-25

**Authors:** Ranga P. Thiruvenkataramani, Amal Abdul-Hafez, Tulasi Kesaraju, Hend Mohamed, Sherif Abdelfattah Ibrahim, Amira Othman, Hattan Arif, Ahmed A. Zarea, Mohammed Abdulmageed, Myrna Gonzalez Arellano, Tarek Mohamed, Masamitsu Kanada, Burra V. Madhukar, Said A. Omar

**Affiliations:** 1 Division of Neonatology, Department of Pediatrics and Human Development, College of Human Medicine, Michigan State University, East Lansing, MI 48824, USA; abdulhaf@msu.edu (A.A.-H.); kesaraj1@msu.edu (T.K.); moham162@msu.edu (H.M.); ibrahi22@msu.edu (S.A.I.); othmana2@msu.edu (A.O.); arifhatt@msu.edu (H.A.); abdulmag@msu.edu (M.A.); gonza990@msu.edu (M.G.A.); mohame54@msu.edu (T.M.); madhukar@msu.edu (B.V.M.); omar@msu.edu (S.A.O.); 2 Regional Neonatal Intensive Care Unit, University of Michigan Health-Sparrow Hospital, Lansing, MI 48912, USA; 3 The Institute for Quantitative Health Science & Engineering, Michigan State University, East Lansing, MI 48824, USA; zarea@msu.edu (A.A.Z.); kanadama@msu.edu (M.K.); 4 Department of Pharmacology and Toxicology, College of Human Medicine, Michigan State University, East Lansing, MI 48824, USA

**Keywords:** small extracellular vesicles, sEVs, extracellular vesicles, exosomes, lung injury, MSCs-derived small extracellular vesicles, mesenchymal stem cells, exosomes, cord blood plasma

## Abstract

Sepsis is a risk factor associated with increasing neonatal morbidity and mortality, acute lung injury, and chronic lung disease. While stem cell therapy has shown promise in alleviating acute lung injury, its effects are primarily exerted through paracrine mechanisms rather than local engraftment. Accumulating evidence suggests that these paracrine effects are mediated by mesenchymal stem cell (MSC)-derived small extracellular vesicles (sEVs), which play a critical role in immune system modulation and tissue regeneration. sEVs contain a diverse cargo of mRNA, miRNA, and proteins, contributing to their therapeutic potential. We hypothesize that sEVs derived from three distinct sources, cord blood plasma (CBP), Wharton jelly (WJ), and placental (PL) MSCs, may prevent the cytotoxicity induced by *E. coli* lipopolysaccharide (LPS) in lung alveolar epithelial cells. Objective: To determine the effects of CBP-, WJ-, and PL-MSCs-derived sEVs on cell viability, apoptosis, and proinflammatory cytokine production in alveolar epithelial cells and monocytes following LPS treatment. sEVs were collected from conditioned media of PL-MSCs, WJ-MSCs, and CBP using 50 nm membrane filters. sEVs were characterized based on nanoparticle tracking analysis (NTA), transmission electron microscopy (TEM), and Western blotting techniques. The protein concentration of isolated sEVs was used to standardize treatment doses. A549 cells and monocyte THP-1 cells were cultured and exposed to LPS in the presence or absence of sEVs for 72 h. Cell viability was measured using CellTiter-Glo 2.0 chemiluminescence-based assay. For cytokine analysis, A549 and THP-1 cells were pre-incubated for 24 h with or without PL- and CBP-sEVs, followed by exposure to LPS or control conditions for an additional 24 h. The conditioned media were collected, and interleukin-6 (IL-6) and interleukin-8 (IL-8) levels were quantified using ELISA. LPS treatment significantly reduced the viability of both A549 and THP-1 cells. The presence of CB- or WJ-sEVs significantly increased cell viability compared to controls. Cells treated with PL-sEVs showed increased cell viability but did not reach statistical significance. LPS-treated cells showed a significant increase in apoptosis and elevated levels of pro-inflammatory cytokines IL-6 and IL-8. All three sEVs types (CBP-, WJ-, and PL-sEVs) significantly reduced LPS-induced apoptosis and IL-6 release. Interestingly, while WJ-sEVs decreased IL-8, both CBP- and PL-sEVs led to an increase in IL-8 compared to their respective controls. CBP-, PL-, and WJ-derived sEVs demonstrated protective effects against LPS-induced injury in alveolar epithelial cells and monocytes, as evidenced by increased cell viability and modulation of pro-inflammatory cytokine release. These findings suggest that placenta-derived sEVs have the potential to modulate the immune response, mitigate inflammation, and prevent end-organ damage in neonatal sepsis.

## 1. Introduction

Bronchopulmonary dysplasia (BPD) is the most prevalent and an important sequela of preterm birth with significant long-term morbidity. The incidence of BPD in the United States is 10,000 to 150,000 per year that includes nearly 50% of infants with birth weight less than 1000 g [1,2]. BPD predisposes infants to prolonged initial hospitalization and increased rates of mortality and childhood morbidity [3,4]. During childhood, long-term morbidities, such as growth failure, chronic respiratory, cardiovascular, and neurodevelopmental impairments, are more common in preterm infants with BPD [4,5,6,7,8,9]. BPD remains an important cause of mortality and long-term respiratory morbidities, such as reactive airway diseases, abnormal pulmonary function, and low exercise capacity [10]. BPD is a chronic lung disease primarily seen in premature infants needing prolonged oxygen supplementation and ventilator support. The risk and severity of developing BPD is inversely related to the extent of immaturity [11]. BPD is a multifactorial disease, and potential causes include oxygen toxicity, ventilator-associated injury, chorioamnionitis, infection, and an immature immune system [12]. No effective therapy to date has been identified and supportive care and gentle ventilation are the current recommendations.

Chorioamnionitis is associated with preterm labor and intrauterine fetal inflammatory response and may promote an inflammatory state and is associated with lung injury in preterm neonates [13]. In ventilated preterm infants, chorioamnionitis is associated with increased lung type IV collagenase levels. Up-regulation of matrix metalloproteinase-9 (MMP-9), a type IV collagenase, associated with antenatal lung inflammation, may play an important role in the pathogenesis of BPD [14]. Prenatal in utero inflammatory responses, such as chorioamnionitis, which have shown increased lung inflammation from postnatal day one, may cause lung injury and eventually development of BPD in mechanically ventilated preterm infants [15]. Antenatal exposure to cytokines, interleukin-6 [IL-6], interleukin-8 [IL-8], tumor necrosis factor-alpha [TNF-a], interleukin-1beta [IL-1b], is a risk factor for developing BPD and might predispose the subset of neonates exposed to chorioamnionitis prenatally to the development of BPD [16,17].

Recent research has shown that stem cells, including mesenchymal stromal cells (MSCs) and hematopoietic stem cells (HSCs) derived from various sources (e.g., adipose tissue, dental pulp, amniotic membrane, Wharton jelly, umbilical cord blood) have demonstrated promising potential as a treatment for tissue regeneration [18,19,20]. The potential mechanism by which stem cells exert their regenerative effects is local tissue engraftment and their paracrine effect via growth factors, cytokines, and extracellular vesicles (EVs) [21,22,23]. The therapeutic efficacy of stem cells is largely attributed to the production of EVs, which carry a diverse array of macromolecules as their cargo.

EVs are small lipid bi-layered membrane-enclosed vesicles released by cells into the extracellular space [24]. EVs play a key role in cellular communication and regulation of cellular responses. EVS carry a unique set of secretomes as a part of their bioactive cargo that reflects the source cells. Upon their extracellular release, EVs modulate the target cells and processes such as coagulation, inflammation, apoptosis, angiogenesis, and proliferation [25]. EVs can be isolated from a wide array of biological fluids (e.g., plasma, urine, broncho-alveolar lavage, saliva, sputum, and pleural effusions). The levels of EVs and molecular cargo are altered based upon the cellular source in different disease states. This suggests that EVs could serve as biomarkers, injurious stimuli, and potential therapeutic targets [25]. EVs can be classified according to their size and biogenesis. The EVs are primarily categorized based on their size as exosomes (~30–150 nm), microvesicles (MVs), also named microparticles (MPs) or ectosomes (~0.1–1 μm), and apoptotic bodies (ABs) (~2–5 μm) [25,26,27]. The International Society for Extracellular Vesicles (ISEV) recommends the use of the generic term ‘EV’ or sEVs instead of exosomes to avoid misleading terms [28]. Exosomes were initially thought to be cellular waste, but recently are thought to play a role in intercellular communication and may play an important role in the disease process with their DNA and miRNA load. The molecular cargo of EVs varies depending on the cellular origin and specific conditions under which EVs were stimulated and released. Overall, the bioactive molecular cargo of EVs is composed of proteins, nucleic acids (including DNA, mRNA, microRNA [miRNA], and long noncoding RNA [lncRNA]) and lipids. EVs can also carry whole organelles, such as mitochondria [29,30]. One of the proposed mechanisms of action for Wharton jelly-derived exosomes is via macrophage immunomodulation and paracrine effect [31,32].

To our knowledge, no previous study has compared the efficacy of small extracellular vesicles (sEVs) from different placental regions and cord blood plasma (CBP) in ameliorating the disease processes. This is the first study to report the protective effects of placental-derived sEVs in the setting of acute lung injury.

## 2. Results

### 2.1. Characteristics of sEVs from Cord Blood Plasma (CBP)-, Placental-, and Wharton Jelly-MSCs

**Figure 1:**
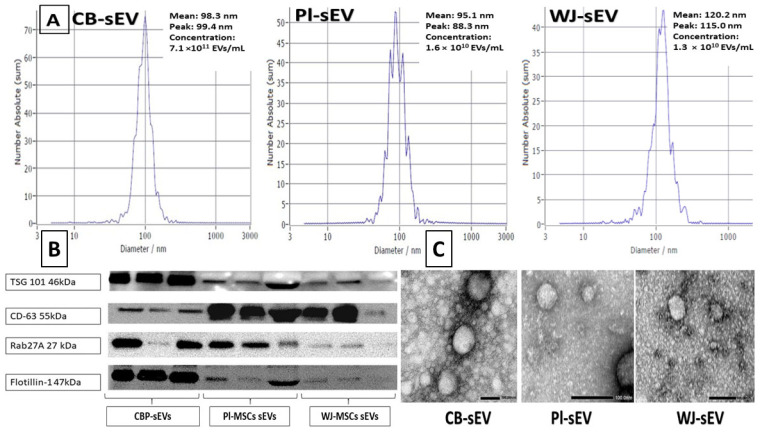
Particle size distribution, morphology, and presence of sEVs surface markers in the isolated CBP-, PL-, and WJ-sEVs. (**A**): Characterization of isolated sEVs using nanoparticle tracking. NTA was carried out using the Zeta View (Particle Metrix, Inning am Ammersee, Germany). The size distribution for the isolated placental derived sEVs (PL, WJ) corresponds to the known size of sEVs, with peaks around 100 nm in diameter. (**B**): Detection of sEVs surface markers in isolated placental sEVs. Equal sEVs protein mass was used for the Western blotting using TSG101, CD63, Rab27A, and Flotillin-1 sEVs markers antibodies. ECL detection and imaging on ChemiDoc MP System was performed (Bio-Rad). The sEVs markers were successfully detected in both WJ-sEVs and PL-sEVs. (**C**): Visualization of the sEVs using electron microscopy (magnification 100.00 nm).

The characteristics of the isolated placental-sEVs are shown in Figure 1. Nanoparticle tracking analysis (NTA) showed the size distribution for the isolated placental-derived sEVs from all three sources (CBP, PL, and WJ) was consistent with the known size of sEVs, with peaks around 100 nm in diameter (Figure 1A). Surface markers were detected in the isolated placental sEVs by Western blotting. All three sources of sEVs showed immunoreactive bands for TSG101, CD63, Rab27A, and Flotillin-1 sEVs markers antibodies (Figure 1B). The morphology and size were confirmed by visualization of the sEVs using electron microscopy imaging showing the particle size and morphology (Figure 1C).

### 2.2. Cytotoxicity Assay/Dose Response Curve

**Figure 2:**
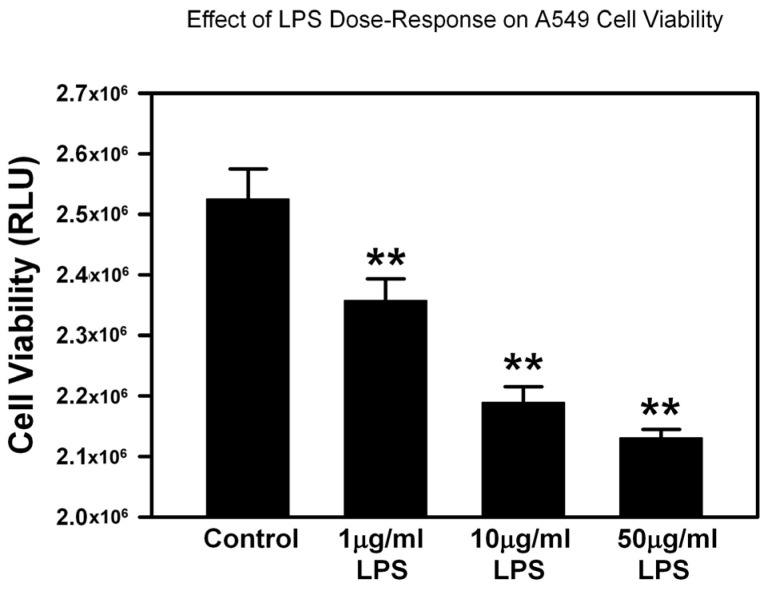
Dose-response effects of LPS on A549 cell viability. A549 cells were exposed to LPS at different concentrations. Cell viability was measured after 48 h using CellTiter Glo 2.0. ** *p* < 0.01 vs. control by ANOVA and post-test.

LPS significantly decreased A549 alveolar epithelial cells viability in a dose-dependent manner when cells were incubated with 1, 10, or 50 µg/mL LPS, as seen in Figure 2. The LPS dose of 10 µg/mL was chosen as the optimal concentration for the experiments with A549 cell and THP-1 cells and the same dose was used to maintain the same experimental conditions. 

### 2.3. Cell Viability Results

**Figure 3:**
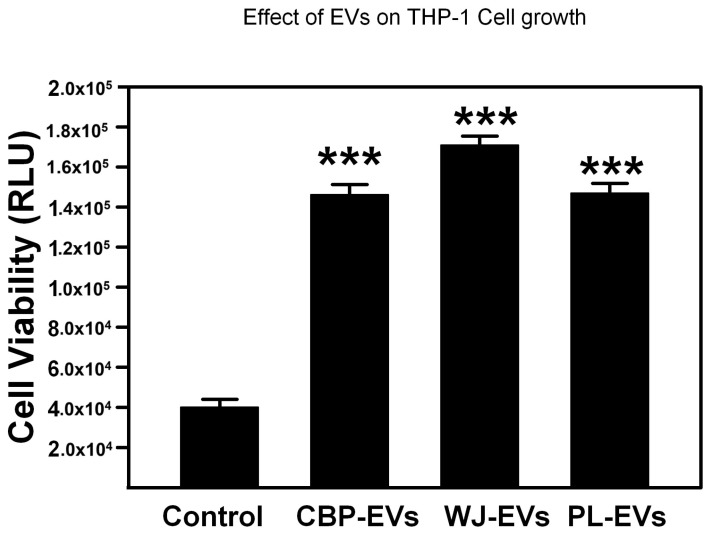
Effects of placental-derived sEVs on THP-1 cell growth. THP-1 cells were cultured in media after adding 400 µg/mL of sEVs for 72 h. Cell growth was measured by detecting cell viability by Cell Titer Glo. *** *p* < 0.001 vs. control by ANOVA and post hoc test.

All three sources of isolated sEVs significantly increased cell viability. In THP-1 monocytes cultured in the presence of the three isolated sEVs, there was a significant increase in cell number compared with the control, as seen in Figure 3.

### 2.4. Placental-Derived sEVs Increase Cell Viability in LPS-Treated Cells

**Figure 4:**
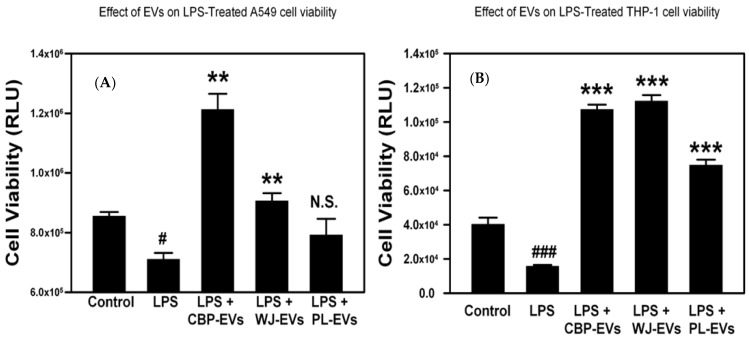
Effects of placental-derived sEVs on restoration of cell viability in LPS-treated cells. Alveolar epithelial A549 cells (**A**) or THP-1 monocytes (**B**) were cultured in media +/− placental-derived sEVs then treated +/− LPS for 72 h. Cell viability was measured by CellTiter Glo kit (Promega, Madison, WI, USA). # *p* < 0.05, ### *p* < 0.001 vs. Control, ** *p* < 0.01, *** *p* < 0.001, N.S. not significant vs. LPS by ANOVA and post-test.

**Figure 5:**
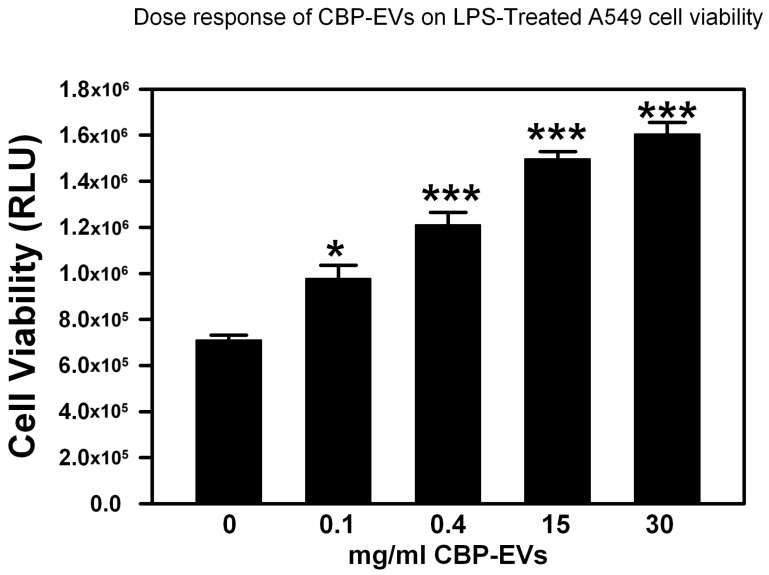
Effect of different doses on restoration of cell viability in LPS-treated cells. Alveolar epithelial A549 cells were treated with increasing concentrations of CBP-EVs and then treated with LPS for 72 h. Cell viability was measured by CellTiter Glo kit. * *p* < 0.05, *** *p* < 0.001.

While LPS significantly decreased both A549 and THP-1 viability, treatment of cells with the isolated placenta-derived sEVs antagonized this effect, as seen in Figure 4. Isolated sEVs from CB and WJ sources significantly increased LPS-treated A549 cell viability, while PL-sEVs showed a trend of restoring A549 cell viability; however, it was not statistically significant (Figure 4A). On the other hand, all three sources of the isolated placental-derived sEVs (CBP, WJ, and PL), significantly increased cell viability after exposure to the toxic effect of LPS (Figure 4B). The degree of restoration of cell viability was shown to be directly proportional to the sEVs treatment concentration in A549 treated with CB-sEVs and LPS (Figure 5). The restoration of viability lost by LPS treatment was also consistent among different individual placental donors.

### 2.5. Necrosis and Apoptosis Assay (A549 Cells and THP-1 Cells)

**Figure 6:**
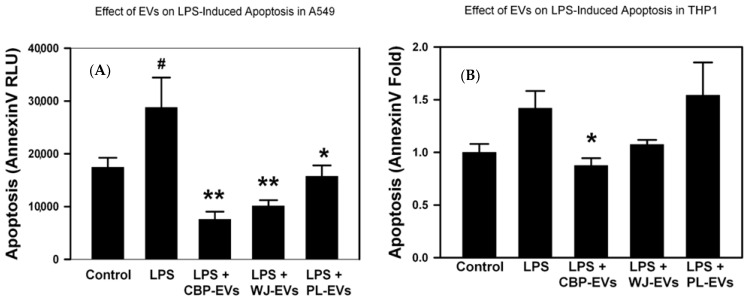
Effect of placental-derived EVs on apoptosis in cells treated with LPS. Alveolar epithelial A549 cells (**A**) or THP-1 monocytes (**B**) were cultured in media +/− placental-derived EVs then treated +/− LPS for 24 h. Annexin V apoptosis was measured by RealTime-Glo™ Annexin V kit. # *p* < 0.05, vs. Control, * *p* < 0.05, ** *p* < 0.01, vs. LPS by ANOVA and post-test.

To examine the cell-type-dependent effects of all three sources of placental sEVs on apoptosis, treatment with LPS induced apoptosis in A549 alveolar epithelial cells, and co-treatment with placental sEVs reversed this effect and showed a significant decrease in apoptosis detected by annexin V (Figure 6A). In THP-1 monocytes, the increase in apoptosis by LPS was not statistically significant (Figure 6B). Only CBP-derived sEVs showed a statistically significant decrease in annexin V when compared with LPS-treated THP-1 monocytes (Figure 6B).

### 2.6. Cytokine Assay Results (A549 and THP-1 Cells)

**Figure 7:**
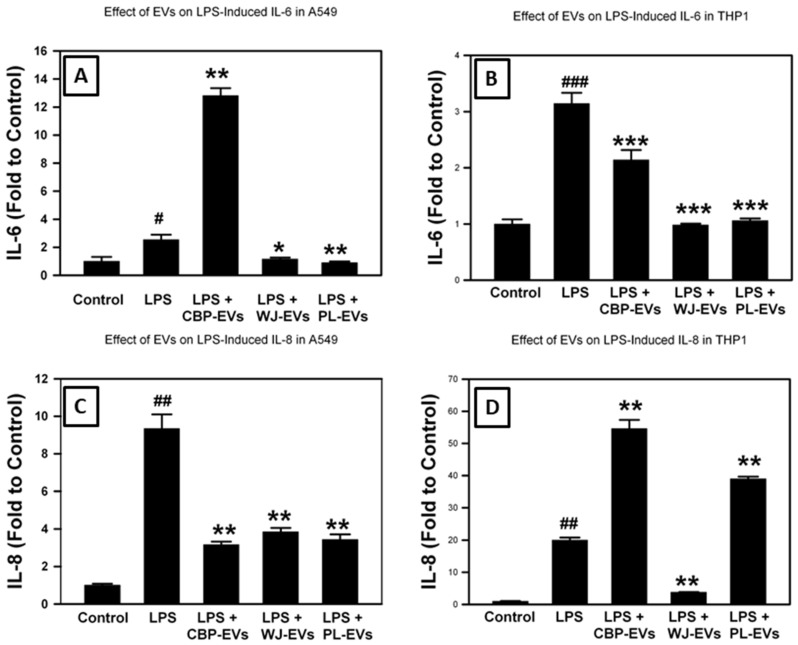
Effect of placental-derived EVs on LPS-induced inflammatory cytokine release. Alveolar epithelial A549 cells (**A**,**C**) or THP-1 monocytes (**B**,**D**) were cultured in media +/− placental-derived EVs then treated +/− LPS for 24 h. ELISA for IL-6 (**A**,**B**) and for IL-8 (**C**,**D**) was performed on cell culture supernatants. Levels of released cytokines after LPS-treatment were expressed as fold to the corresponding control. # *p* < 0.05, ## *p* < 0.01, ### *p* < 0.001 vs. Control, * *p* < 0.05, ** *p* < 0.01, *** *p* < 0.001 vs. LPS by ANOVA and post-test.

To examine the effect of all three sources of placental sEVs on LPS-induced cytokine release, A549 and THP-1 cells were treated with LPS. This treatment significantly induced the release of both pro-inflammatory cytokines IL-6 and IL-8 compared with control, as seen in Figure 7. Only WJ- and PL-sEVs significantly decreased LPS-induced IL-6 release in A549, while CB-sEVs significantly increased the LPS-induced IL-6 release (Figure 7A). In THP-1 cells, all sEVs sources significantly decreased LPS-induced IL-6 release (Figure 7B). In A549 cells, all sEVs sources significantly decreased LPS-induced IL-8 release (Figure 7C). Only WJ-sEVs significantly decreased LPS-induced IL-8 release in THP-1, while CB- and PL-sEVs significantly increased the LPS-induced IL-8 release (Figure 7D).

## 3. Discussion

Inflammation plays a key role and is a major contributing factor in acute lung injury and disordered lung repair leading to the development of BPD [33]. Chorioamnionitis causes a significant in utero inflammatory response in the fetus and postnatal inflammatory sequelae, and this plays an important role in the pathogenesis of BPD, especially in a premature infant. This lung injury can be secondary to inflammatory response, oxidative stress, and progression to fibrosis [15,16,17]. Inflammation could potentially lead to necrosis of epithelial cells, fibrosis and scarring of lung tissue, abnormal septation and alveolar simplification, and dysregulation of microvascular growth and maturation [34,35,36].

LPS exposure has been shown to cause decrease in cell viability. In this study, we have shown that small EVs derived from cord blood plasma, Wharton jelly and placental MSCs improved the cell viability or rescued the cells from LPS-induced cell injury in A549 and THP-1 cells. Liu et al. has presented results that exosomal miR-132-3p derived from MSCs mediated protective effects on LPS-induced acute lung injury [37]. Exosomal miR-132-3p potentiated cell proliferation and suppressed apoptosis in LPS-induced MLE-12 cells by targeting TRAF6 and inactivating PI3K/Akt signaling [37].

Cui et al. have demonstrated that alveolar epithelial cell injury (AECs) and acute lung injury induced by LPS lead to cell apoptosis, inflammatory changes, and oxidative stress via the Toll-like receptor (TLR) 2/activator protein-1 (AP-1) pathway [38]. In this study, we demonstrated that treatment with small EVs derived from cord blood plasma, Wharton jelly and placental MSCs in LPS-exposed lung epithelial cells alleviated apoptosis in A549 cells and THP-1 cells. Shen et al. have shown miR-125b-5p in adipocyte stem cells (ADSCs)-derived exosomes alleviate pulmonary microvascular endothelial cells ferroptosis in sepsis-induced lung injury [39]. ADSCs-derived exosomes have been shown to enhance both the expression and nuclear translocation of Nrf2, while simultaneously reducing the expression of Keap1. Specifically, these exosomes facilitate the delivery of miR-125b-5p, which alleviates the oxidative stress by inhibiting Keap1 and suppressing ferroptosis via the Keap1/Nrf2/GPX4 signaling axis [39]. In sepsis-induced lung injury, ADSCs exosomes demonstrated a protective effect by inhibiting ferroptosis and up-regulating GPX4 expression in pulmonary microvascular endothelial cells.

EVs have both pro-inflammatory and anti-inflammatory roles, and their effect depends on the origin cell type and stage of inflammation [40]. Shen et al. have shown exosomes from adipocyte-derived stem cells (ADSCs) alleviated LPS-induced inflammation and oxidative stress. ADSCs exosomes play a role in the polarization of macrophages to an anti-inflammatory phenotype, which, in turn, up-regulate HO-1 and Nrf2 and down-regulate Keap1. The expression of the inflammatory cytokines IL-1β, TNF-α, and IL-6 in macrophages was down-regulated secondary to the nuclear translocation [31,41]. Willis et al. also showed Wharton jelly-MSCs-derived exosomes ameliorated experimental BPD and restored lung function through macrophage immunomodulation by promoting macrophage polarization from M1 pro-inflammatory to M2 anti-inflammatory [31]. Wang et al. also showed that gingival-MSCs-derived exosomes promoted macrophage polarization from M1 pro-inflammatory to M2 anti-inflammatory under inflammatory conditions [42]. Our research revealed distinct effects of small EVs from various sources on inflammatory cytokine expression in A549 and THP-1 cells. EVs derived from Wharton jelly MSCs down-regulated both inflammatory cytokines IL-6 and IL-8 in A549 and THP-1 cells. In contrast, sEVs derived from cord blood plasma were associated with a decrease in IL-6 while increasing IL-8 in THP-1 cells. Placental-MSCs-derived EVs demonstrated a complex pattern of cytokine modulation. They down-regulated IL-6 and IL-8 in A549 cells, while in THP-1 cells, they down-regulated IL-6 but up-regulated IL-8. These findings highlight the source-dependent and cell-type-specific effects of EVs on inflammatory cytokine regulation. Cytokines play a key role in cellular communication. We speculate that EVs carry cytokines in their cargo and on their surface [43].

The transcription factor nuclear factor-kB (NF-kB) plays a vital role in the signaling pathway in all nucleated cells by regulating genes associated with inflammation and is also involved in multiple cellular responses to chemical or physical stimuli [44]. NF-κB is the primary regulator of the senescence-associated secretory phenotype (SASP), which consists of inflammatory cytokines (interleukin [IL]-6 and IL-8), proteases (matrix metalloproteinases), chemokines (monocyte chemoattractant proteins and macrophage inflammatory proteins), and growth factors (granulocyte–macrophage colony-stimulating factor and transforming growth factor-β), and has a significant function in aging [45,46]. Tu et al. showed exosomes derived from endothelial cells contain heat-shock protein A12B, and down-regulated TNF-a and IL-1b production and up-regulated IL-10 production in LPS-stimulated macrophages. It was shown that exosomes down-regulate NF-κB activation and nuclear translocation in LPS-stimulated macrophages [47]. Exosomes derived from human umbilical cord MSCs [47] and murine bone marrow derived-MSCs [48] after pretreatment with IL-1β effectively enhanced the immunomodulatory properties of MSCs, partially through the exosome-mediated transfer of miR-146a and miR-21, respectively, in cecal ligation and puncture (CLP) septic models and improved their survival in septic mice [48,49].

A limitation of our study is that all the experiments were conducted on A549 and THP-1 cell lines. We were able to test the effect of sEVs on proinflammatory cytokines (IL-6 and IL-8) but were not able to test the anti-inflammatory cytokines (IL-10) because A549 cells do not express IL-10. The in vitro study is a first step to study the efficacy of the therapeutic potential of sEVs in experimental conditions. Our next steps are to conduct experiments in an in vivo model and to test the effect of sEVs on the cytokine panel.

Our in vitro study, as well as many published reports, clearly suggest that sEVs are a valuable therapeutic modality for the treatment of sepsis-induced lung injury, as well as other pathological and disease conditions. However, our study also points to the fact that there is considerable heterogeneity in sEVs that could impact their clinical use. In fact, sEVs heterogeneity imposes a major limitation in the clinical use of sEVs for therapeutic purposes as highlighted in some recent reviews [50,51,52,53,54]. The heterogeneity among the different subpopulations of sEVs arises due to the nature of their biogenesis and the cargo they contain. This heterogeneity continues to be a challenge in the use of sEVs as a therapeutic modality for personalized medicine in clinical settings [50,51,52,53,54]. Future research efforts should focus on separating the different subpopulations of sEVs and characterizing their cargo.

## 4. Materials and Methods

### 4.1. Institutional Review Board (IRB) and Maternal Consent

The research related to human subject use has complied with all the relevant national regulations and institutional policies and is in accordance with the tenets of the Helsinki Declaration and has been approved by the institutional review boards at Michigan State University, East Lansing, Michigan and Sparrow Hospital, Lansing, MI, USA [33]. Informed consent was obtained from the mothers of all study subjects before the UCB, Wharton jelly and placenta were collected from healthy full-term (FT) pregnancies with no evidence of morbidities such as maternal hypertension, diabetes mellitus (DM) or fetal growth restriction.

### 4.2. Cell Culture

The following cell lines were used for our in vitro cultures.

#### 4.2.1. Human Lung Adenocarcinoma (A549) Cell Line

The human lung adenocarcinoma A549 cell line was obtained from the American Type Cell Culture Collection (ATCC) and cultured in Ham’s F12 medium (ATCC, Manassas, VA, USA), supplemented with 10% fetal bovine serum (FBS) (Gibco, Grand Island, NY, USA). The cells were grown, maintained, and handled according to the supplier’s manual [12]. All experiments were conducted in a serum-free Ham’s F12 medium. The cells were grown to 70% sub-confluence in FBS-supplemented medium and the medium was replaced with serum-free F12 medium. The cells were preincubated for 24 h in the presence or absence of sEVs at a dose of 400 μg/mL and then incubated with LPS for 24 h for cytokine expression to examine the effect of EVs in prevention of cell injury, and for 72 h to measure cell viability. An equal volume of serum-free media was added in the sEVs-free control group. The supernatants were collected and stored at −20 °C and were later used for cytokine expression via enzyme-linked immunoassay (ELISA).

#### 4.2.2. Human Leukemia Monocyte (THP-1) Cell Line

THP-1 cells were obtained from the American Type Cell Culture Collection (ATCC) and cultured in RPMI-1640 medium (ATCC, Manassas, VA, USA), supplemented with 10% fetal bovine serum (FBS) (Gibco, Grand Island, NY, USA), as described earlier. The cells were grown, maintained, and handled according to the supplier’s manual. All experiments were conducted in a serum-free RPMI-1640 medium. Experimental conditions were the same as described in the prior section.

### 4.3. Isolation and Characterization of sEVs from Cord Blood Plasma, Placental- and Wharton Jelly-MSCs Supernatant

#### 4.3.1. Collection of Cord Blood Plasma and Condition Media (CM) from Placenta (PL) and Whorton Jelly (WJ) Mesenchymal Stem Cells (MSCs)

The EV-depleted fetal bovine serum (FBS) was prepared by ultracentrifugation at 100,000× *g* at 4 °C for 20 h and the EV-depleted FBS was used to prepare complete media. MSCs were seeded at 7 × 10^5^ cells or 1.5 × 10^6^ cells per 60 mm or 100 mm cell culture dishes, respectively. After the cells reached 80% confluence, the regular medium was replaced with EV-depleted medium after washing twice with PBS. The conditioned medium (CM) was collected after 48 h and stored at −80 °C for later sEV extraction as described below.

#### 4.3.2. Isolation of sEVs

The isolation of sEVs from the CBP and CM of PL and WJ MSCs is summarized below. Briefly, aliquoted samples of diluted CBP (diluted 1:3 in PBS) or the supernatant cultured media were briefly centrifuged at 600× *g* for 10 min at room temperature to remove the cells and debris. Then, the supernatant was centrifuged at 2000× *g* for 30 min to remove the apoptotic bodies at room temperature. The supernatant was then filtered with 0.22 μm PES membrane filters (Cell pro filters, ThermoFisher, Waltham, MA, USA) with vacuum to remove large EVs (>220 nm). The sEVs were then collected using ultrafiltration technique using a 50 nm membrane filter (Whatman, Maidstone, UK, WHA110603) with holders (EMD Millipore, Burlington, MA, USA, SX0002500). The holders with the filters were attached to the vacuum manifold (Qiagen, Hilden, Germany) and the filter was washed with 10 mL PBS with continuous vacuum. Then, the supernatant was ultrafiltered with 50 nm membrane filter and then with 10 mL serum-free media wash. When the sample reached 200–500 μL above the filter (sEVs with particle size between 50–220 nm), the samples were collected and aliquoted in 1.5 mL microtubes and stored in −80 °C for future use. The protein concentration was measured using Bradford Assay (ThermoFisher, Waltham, MA, USA) [55]. The size range, morphology, and protein markers of the collected sEVs were analyzed by nanoparticle tracking analysis (NTA, ZetaView, Particle Metrix, Meerbusch, Germany), transmission electron microscopy (TEM) (Japan Electron Optics Laboratory, Tokyo, Japan), and Western blotting (WB).

#### 4.3.3. Nanoparticle Tracking Analysis

Nanoparticle tracking analysis (NTA) was carried out using the Zeta View (Particle Metrix, Meerbusch, Germany) following the manufacturer’s instructions. sEVs derived from CBP-, PL-, and WJ-MSCs were further diluted 100- to 1000-fold with PBS for the measurement of particle size and concentration [55].

#### 4.3.4. Western Blotting for sEVS

Equal amounts of total protein (10 ug) derived from CBP-, PL-, and WJ-MSCs sEVs were mixed with 5x Pierce™ Lane Marker Reducing Sample Buffer (Thermo Fisher Scientific, Waltham, MA, USA). The following four primary antibodies were used for the common EVs surface markers using our protocol [33]: TSG101, Rab27a, and Flotillin1 with b-mercaptoethanol and without b-mercaptoethanol for detecting CD63 [55]. Proteins were separated on a 4–20% Mini-PROTEAN TGX gel (Bio-Rad, Hercules, CA, USA) and transferred to a polyvinylidene difluoride membrane (PVDF) (Millipore, IPFL00010). After blocking with 5% ECL Blocking Agent (GE Healthcare, Chicago, IL, USA, RPN2125) at room temperature for 1 h, membranes were probed with primary antibodies overnight at 4 °C at dilutions recommended by the suppliers as follows: TSG 101 (Proteintech, Wuhan, China, 28283-1-AP), Rab27a (Proteintech, 17817), flottilin-1 (BD, 610820), CD63 (Thermo Fisher Scientific, 10628D, Ts63), followed by wash then incubation with horseradish peroxidase (HRP)-conjugated secondary antibodies at room temperature for 1 h. The membranes were visualized with ECL select Western Blotting Detection Reagent (GE Healthcare, RPN2235) on ChemiDoc Imaging System (Bio-Rad Laboratories Inc., Hercules, CA, USA) [55].

#### 4.3.5. Transmission Electron Microscopy

Samples were prepared as previously reported [55,56,57]. Isolated EVs were fixed in 2% paraformaldehyde for 5 min. For negative staining of sEVs, 5 mL of the sample solution was placed on a carbon-coated EM grid and EVs were immobilized for 1 min. The grid was transferred to five drops of distilled water 100 µL each and left on the surface of each drop for 2 min sequentially. The sample was negatively stained with 1% uranyl acetate. The excess uranyl acetate was removed by contacting the grid edge with filter paper and the grid was air dried. The grids were imaged with a transmission electron microscope (JEOL100CXII, Japan Electron Optics Laboratory, Japan) operating at 100 kV. Images were captured on a Gatan Orius Digital Camera (Gatan, Inc., Pleasanton, CA, USA).

### 4.4. Cytotoxicity Assay

Cytotoxicity was measured by CellTiter-glo luminescence assay. A549 and THP-1 cells were seeded onto 96-well plates. Cell viability was measured using a CellTiter-Glo^®^ 2.0 Cell Viability Assay kit (Promega, Madison, WI, USA) after 72 h of incubation as per the manufacturer’s instructions and the luminescence was measured using a Biotek Synergy Neo microplate reader (Agilent, Santa Clara, CA, USA). To assess the LPS effect on A549 cells viability, A549 cells were incubated with increasing concentration of LPS (0, 1, 10, 50 μg/mL). THP-1 cells were exposed to sEVs derived from CBP-, PL-, and WJ-MSCs to assess the effect of sEVs on THP-1 cells viability. Cell viability of both A549 and THP-1 cells were assayed in the presence of sEVs from all three sources to assess the effect of 10 μg/mL LPS treatment. The dose of 400 μg/mL of protein concentration of the sEVs (exosomes) was chosen after dose escalation experiment with 100 μg/mL, 200 μg/mL, 400 μg/mL, and 800 μg/mL. The dose of 400 μg/mL gave the optimal results at the lowest dose. 

### 4.5. Cytokine Assay—ELISA Method for Measuring IL-6 and IL-8 Levels

The levels of IL-6 and IL-8 were determined by ELISA kits (Protein tech, San Diego, CA, USA) according to the manufacturer’s instructions. Standard curves were plotted to calculate the concentrations of these inflammatory cytokines in the samples. The manufacturer’s instructions were followed, and the optical density (OD) was measured at a wavelength of 450 nm using a Biotek Synergy Neo microplate reader (Agilent, USA).

### 4.6. Necrosis and Apoptosis Assay (Annexin/PI Assay Kit)

The Real Time-Glo Annexin V apoptosis and necrosis assay (Promega) was used. A549 and THP-1 cells were plated and preincubated with sEVs for 24 h and then were exposed to LPS, and apoptosis and necrosis were measured after 72 h. The manufacturer’s instructions were followed, and the luminescence was measured using a Biotek Synergy Neo microplate reader (Agilent, USA).

### 4.7. Statistics

The results of the study were analyzed using SigmaPlot statistical software version 14.5 (Systat software Inc., San Jose, CA, USA) by the Student’s *t*-test or analysis of variance (ANOVA) to evaluate statistical significance between means. All pairwise multiple comparisons were performed using the Student–Newman–Keul method. Results were statistically significant if *p*-value < 0.05. Results were reported as mean ± SD or mean ± SEM.

## 5. Conclusions

Small EVs derived from placental sources (placenta- and Wharton jelly-MSCs) and cord blood plasma improved cell viability and alleviated lung injury in lung epithelial cells, and down-regulated inflammatory markers secondary to lung injury induced by LPS. The mechanism by which human placental small EVs work is uncertain and to be determined, but human placental-MSCs-derived small EVs may play a therapeutic role in alleviating lung injury and preventing BPD in premature neonates.

## Data Availability

The authors have full control of the primary data, and the authors agree to allow the journal to review their data if requested. All data and material used for writing the manuscript are available.

## Abbreviations

Mesenchymal Stem Cells, MSCs; Extracellular vesicles, EVs; Small Extracellular Vesicles (exosomes), sEVs; Lipopolysaccharide: LPS, Cord Blood Plasma, CBP.
